# The Sedating Antidepressant Trazodone Impairs Sleep-Dependent Cortical Plasticity

**DOI:** 10.1371/journal.pone.0006078

**Published:** 2009-07-01

**Authors:** Sara J. Aton, Julie Seibt, Michelle C. Dumoulin, Tammi Coleman, Mia Shiraishi, Marcos G. Frank

**Affiliations:** Department of Neuroscience, School of Medicine, University of Pennsylvania, Philadelphia, Pennsylvania, United States of America; INSERM U862, France

## Abstract

**Background:**

Recent findings indicate that certain classes of hypnotics that target GABA_A_ receptors impair sleep-dependent brain plasticity. However, the effects of hypnotics acting at monoamine receptors (*e.g.,* the antidepressant trazodone) on this process are unknown. We therefore assessed the effects of commonly-prescribed medications for the treatment of insomnia (trazodone and the non-benzodiazepine GABA_A_ receptor agonists zaleplon and eszopiclone) in a canonical model of sleep-dependent, *in vivo* synaptic plasticity in the primary visual cortex (V1) known as ocular dominance plasticity.

**Methodology/Principal Findings:**

After a 6-h baseline period of sleep/wake polysomnographic recording, cats underwent 6 h of continuous waking combined with monocular deprivation (MD) to trigger synaptic remodeling. Cats subsequently received an i.p. injection of either vehicle, trazodone (10 mg/kg), zaleplon (10 mg/kg), or eszopiclone (1–10 mg/kg), and were allowed an 8-h period of post-MD sleep before ocular dominance plasticity was assessed. We found that while zaleplon and eszopiclone had profound effects on sleeping cortical electroencephalographic (EEG) activity, only trazodone (which did not alter EEG activity) significantly impaired sleep-dependent consolidation of ocular dominance plasticity. This was associated with deficits in both the normal depression of V1 neuronal responses to deprived-eye stimulation, and potentiation of responses to non-deprived eye stimulation, which accompany ocular dominance plasticity.

**Conclusions/Significance:**

Taken together, our data suggest that the monoamine receptors targeted by trazodone play an important role in sleep-dependent consolidation of synaptic plasticity. They also demonstrate that changes in sleep architecture are not necessarily reliable predictors of how hypnotics affect sleep-dependent neural functions.

## Introduction

Behavioral findings in animals and humans suggest an important role for sleep in the consolidation of learning and memory; however, much less is known about how sleep affects the synaptic and brain system-level changes that underlie these processes [1], [2]. Certain hypnotic drugs can cause anterograde amnesia during wakefulness [3], [4], and may inhibit synaptic plasticity *in vitro* [5], [6], but it is unclear how they affect sleep-dependent consolidation processes and *in vivo* synaptic plasticity. Importantly, the most commonly-prescribed hypnotics target diverse neurotransmitter systems that may interfere with plastic processes that occur during sleep.

Ocular dominance plasticity in the primary visual cortex (V1) is triggered by monocular deprivation (MD) during a critical developmental window. We have previously shown that the effects of MD are consolidated by subsequent sleep, but inhibited by sleep deprivation, or when sleep is combined with the non-benzodiazepine hypnotic zolpidem [7]–[11]. This suggests that certain classes of hypnotics targeting the GABA_A_ receptor impair synaptic remodeling during sleep. What has not been investigated, however, are the effects of other “z” hypnotics (*e.g.*, zaleplon, [es]zopiclone) [12], [13] and the atypical, but commonly prescribed hypnotic trazodone on sleep-dependent brain plasticity.

In contrast to benzodiazepine and non-benzodiazepine hypnotics, the sedating antidepressant trazodone acts as both a weak serotonin (5-HT) reuptake inhibitor and as an antagonist at 5-HT_2A_ and 5-HT_2C_, α_1_-adrenergic, and histamine H_1_ receptors [14], [15]. Because intracellular signaling pathways regulated by monoaminergic neurotransmission have been shown to modulate diverse forms of *in vivo* synaptic plasticity [16], [17], it is possible that antagonizing monoaminergic signaling with trazodone during sleep inhibits plasticity. To investigate this possibility, we compared the effects of trazodone (TRA), zaleplon (ZAL) and eszopiclone (ESZ) on a classic *in vivo* form of cortical plasticity that is consolidated by sleep.

## Results

### Effects of hypnotics on post-MD sleep architecture and EEG activity

**Figure 1:**
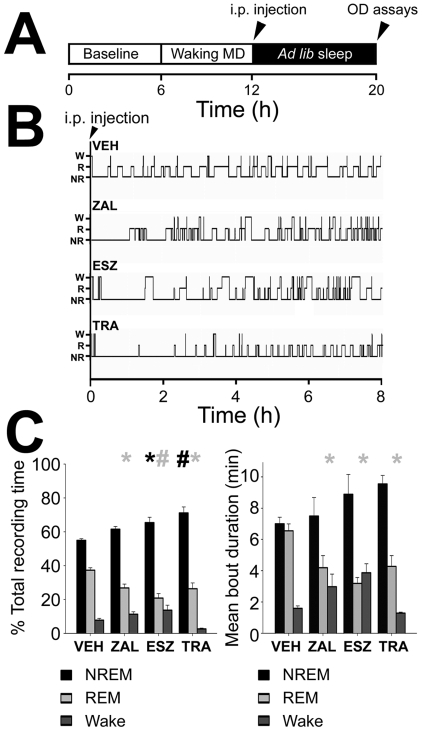
Experimental design and hypnotic effects on post-MD sleep. Schematized experimental design is shown in A. All animals underwent polysomnographic recording over a 6-h baseline period prior to a 6-h period of waking combined with MD. After the MD period, cats received an i.p. injection of either hypnotic or vehicle (time indicated by arrowhead) and were then allowed and 8-h period of *ad lib* sleep in total darkness, after which ocular dominance (OD) was assessed. Representative post-MD hypnograms (B) from animals in each of the treatment groups show transitions between wake (W), REM (R), and NREM (N) sleep. VEH = vehicle (*n* = 6), ZAL = zaleplon (*n* = 5), ESZ = eszopiclone (*n* = 6), TRA = trazodone (*n* = 5). Bar graphs in C show mean (±SEM) % of total recording time (%TRT; left) spent in NREM (black bars), REM (light gray), and wake (dark gray), and mean (±SEM) bout duration for each vigilance state (right) during the post-MD period. * indicates *p*<0.05, # indicates *p*<0.001, vs. VEH, *post hoc* Holm-Sidak or Dunn's test.

Polysomnography showed that the three hypnotics had similar effects on overall sleep/wake amounts and durations (**Fig. 1**). All three drugs led to significant decreases in rapid eye movement (REM) sleep, and concomitant increases in non-REM (NREM) sleep, relative to vehicle (VEH; **Fig. 1C**; effects of treatment on % of total recording time for the three vigilance states: *F* = 6.3, *p* = 0.004 for NREM; *F* = 7.1, *p* = 0.003 for REM; *H* = 12.5, *p* = 0.006 for wake, one-way ANOVA). Similar effects were seen for mean REM sleep bout durations in the post-MD period (**Fig. 1C**), which were significantly reduced in all three hypnotic-treated groups relative to VEH (effects of treatment on REM sleep and wake bout durations: *F* = 6.2, *p* = 0.005, and *H* = 10.5, *p* = 0.015, respectively). Latencies to the first REM sleep episode also increased in all three hypnotic-treated groups, but this was only significant in the ESZ and ZAL-treated cats when compared with VEH cats (latency [mean±SEM]  = 25.6±4.9 min for VEH, 50±14.6 min for TRA, 79.6±9.3 min for ZAL*, 104.3±18.5 min for ESZ*; *F* = 6.5, *p* = 0.004, one-way ANOVA; * indicates *p*<0.05, Holm-Sidak *post hoc* test vs. VEH). As described previously for other hypnotic agents [11], we also occasionally observed a state that appeared intermediate between waking and NREM sleep - described as “NREM-drowsy” (ND) - in a subset of ESZ- and ZAL-treated animals (ESZ, *n* = 2; ZAL, *n* = 1). However, the amount of ND in these animals was very low across the 8-h post-MD sleep period (0.7%±0.4% and 2.1% of total recording time, respectively; data not shown). Because of the similarity of ND to NREM sleep, ND periods were included in calculations of NREM state amounts and bout durations (but were excluded from NREM EEG analysis), as described previously [11].

Of the three drugs tested, TRA led to the most substantial and persistent increases in NREM sleep time relative to VEH, which continued throughout the 8-h post-MD sleep period (**Fig. S1** **,** **Table S1**; TRA vs. VEH: *p*<0.05 at 2–4 h and 4–6 h post-injection, Holm-Sidak *post hoc* test; ZAL and ESZ vs. VEH: *N.S.* at 2–4 h and 4–6 h post-injection).

**Figure 2:**
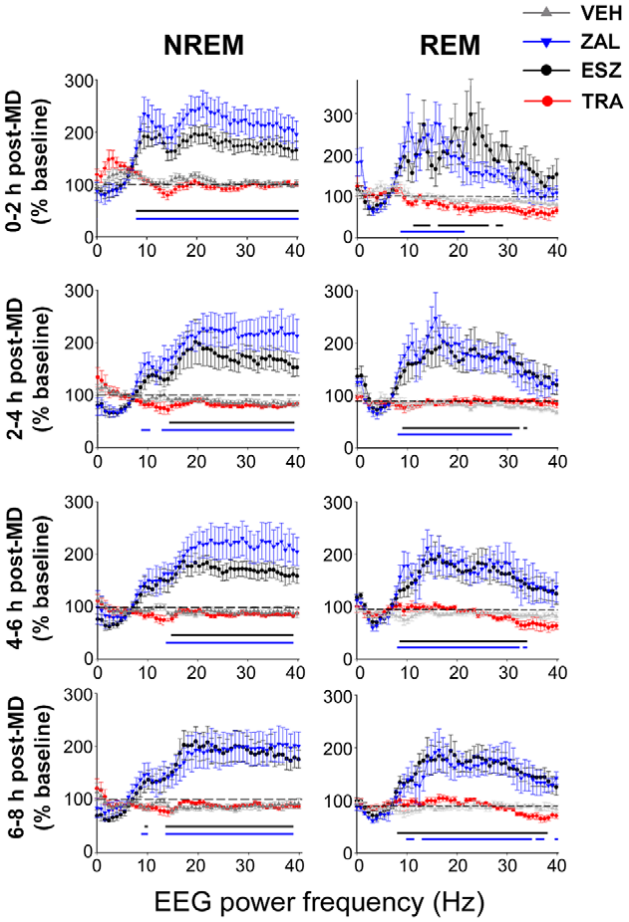
Drug effects on EEG power spectra during post-MD sleep. Data represent NREM and REM EEGs (expressed as a % of corresponding baseline values) averaged in 2-h bins (±SEMs) in the post-MD period. Analysis of variance for EEG power densities during post-MD sleep indicated significant effects of treatment group, and significant group×frequency interactions, throughout the post-MD period (two-way ANOVA, results shown in Table S2). Significant changes in EEG power in hypnotic-treated animals (compared to VEH; Holm-Sidak test vs. VEH, *p*<0.05) are represented by color-coded bars on the bottom of the graphs.

The three hypnotics differed more in their effects on sleeping EEG activity. As previously reported with the benzodiazepine triazolam [11], both ZAL and ESZ led to significant increases over VEH in EEG power spectra in the sigma, beta, and gamma frequency ranges, during both REM and NREM sleep (**Fig. 2** **;** **Table S2**). These changes in EEG activity persisted throughout the post-MD recording period. In contrast, TRA treatment produced no significant changes in EEG power spectra relative to VEH in either sleep state.

### Effects of hypnotics on sleep-dependent cortical plasticity

**Figure 3:**
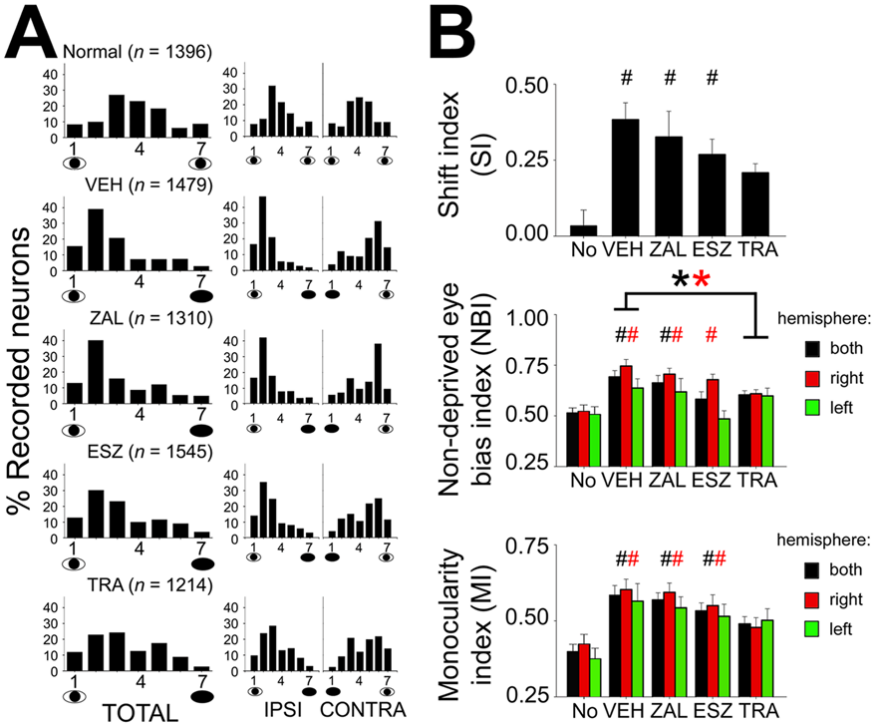
Trazodone impairs sleep-dependent cortical plasticity. Ocular dominance histograms for single neurons (A) recorded from both hemispheres (TOTAL) and in hemispheres ipsilateral (IPSI) and contralateral (CONTRA) to the deprived eye (DE) are shown for each of the treatment groups. Ocular dominance scores were ranked on a 7-point scale as described previously [8]. *n* =  number of neurons recorded in each condition. Quantitative measurements of ocular dominance for both hemispheres are shown in B. One-way ANOVA showed a significant effect of treatment for shift indices (SIs), non-deprived eye bias indices (NBIs), and monocularity indices (MIs). For NBIs, * indicates *p*<0.05, Dunn's and Holm-Sidak test vs. VEH for combined-hemisphere data and right-hemisphere data, respectively. For all measures, # indicates *p*<0.05, Dunn's or Holm-Sidak test vs. Normal (No) values.

Single-unit recordings within the primary visual cortex (V1) showed marked differences in cortical plasticity between the treatment groups. In agreement with an earlier report [11], single-unit recording revealed that animals treated with VEH after the 6-h monocular deprivation (MD) period showed normal ocular dominance plasticity after an 8-h period of post-MD sleep (**Fig. 3A**). While this plasticity was not significantly reduced by ZAL or ESZ treatment, it was inhibited in animals treated with TRA. This inhibition was evident in the ocular dominance distribution of neurons recorded from TRA-treated cats (**Fig. 3A**), and also from scalar measures of ocular dominance (**Fig. 3B**). For example, the overall change seen in SIs in VEH-treated animals (vs. that seen in “Normal” cats [with unmanipulated binocular vision and sleep]) was reduced by 46% when animals were treated with TRA. Moreover, only VEH-, ZAL-, and ESZ-treated cats showed significantly greater SIs than Normal cats (*F* = 6.3, p = 0.001, one-way ANOVA; *p*<0.05, Holm-Sidak test vs. Normal; **Fig. 3B**). Similar effects of TRA were seen for single-unit non-deprived eye bias indices (NBIs; NBI_both hemispheres_: *H* = 18.8, *p*<0.001, NBI_right hemisphere_: *F* = 9.6, *p*<0.001; NBI_left hemisphere_: *N.S.*, one-way ANOVA; **Fig. 3B**). The overall increase in NBI values in VEH-treated cats was significantly reduced in neurons recorded from TRA-treated cats (by 50%; *p*<0.05, Dunn's *post hoc* test vs. VEH). Monocularity indices (MIs) were similarly affected, with an overall reduction of 51% in TRA-treated animals. Again, when Normal data were compared with the four treatment groups, VEH-, ZAL-, and ESZ-treated cats showed significantly greater single-unit MIs (*F* = 8.5, p<0.001, one-way ANOVA; *p*<0.01, Holm-Sidak test vs. Normal), while TRA-treated cats did not (**Fig. 3B**).

Similar trends were observed using intrinsic signal imaging as a second measure of ocular dominance plasticity (with lower overall values in the TRA group; **Text S1**, **Fig. S2**), but these did reach significance when the VEH- and hypnotic-treated groups were compared directly (one-way ANOVA, *N.S.*). However, scalar measures of plasticity were all decreased in TRA-treated V1 (by 13%, 13%, and 58% for SI, NBI, and MI, respectively) compared with changes in VEH-treated animals. When data from the four treatment groups were compared with data from Normal cats (**Fig. S2C**), VEH-, ZAL-, and ESZ-treated cats showed significantly greater intrinsic signal MIs (*H* = 18.6, p<0.001, Kruskall-Wallis one-way ANOVA; *p*<0.05, Dunn's test vs. Normal) - indicative of greater plasticity, while TRA-treated cats did not.

### Effects of hypnotics on response properties of V1 neurons

**Figure 4:**
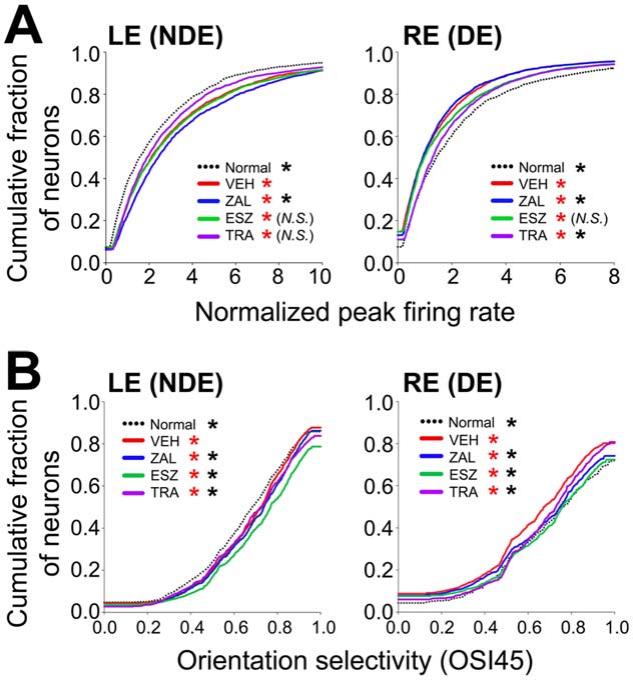
Trazodone impairs sleep-dependent changes in deprived eye and non-deprived eye responses. Cumulative distributions in A show normalized peak firing responses of V1 neurons from each treatment group to preferred-orientation stimulation of the left eye (NDE) and right eye (DE). Data from VEH and hypnotic-treated groups are compared to values from normally-sighted, untreated controls (Normal). All groups showed significant augmentation of NDE firing response rates vs. Normal; responses from ZAL-treated animals showed further enhancement relative to VEH. In contrast, NDE response augmentation was slightly (but not significantly) reduced in V1 of TRA-treated cats (*p* = 0.09 vs. VEH). All groups also showed significant depression of DE responses compared to responses in Normal V1; this depression was significantly inhibited in TRA-treated cats, and was significantly enhanced in ZAL-treated cats (vs. VEH). Cumulative distributions in B show orientation selectivity indices (OSI45) for the two eyes, assessed as described above. In all groups, NDE orientation selectivity was enhanced relative to that seen in Normal V1. NDE selectivity was further enhanced (relative to VEH) in neurons from ESZ- or ZAL-treated cats, and slightly (but significantly) reduced in neurons from TRA-treated cats. All groups also showed reduced DE orientation selectivity (relative to Normal V1). In all hypnotic-treated groups, DE selectivity was significantly less depressed than in neurons from VEH-treated cats. * in red indicates *p*<0.05 vs. Normal, Kolmogorov-Smirnov [K-S] test. * in black indicates *p*<0.05 vs. VEH, K-S test.

We analyzed additional single-unit response properties in neurons from the main treatment groups to further define changes in non-deprived eye (NDE) and deprived eye (DE) pathways. These included comparisons of normalized spike rates at the preferred stimulus orientation for both eyes (**Fig. 4A**; see **MATERIALS AND METHODS** for description of normalization procedures) and assessments of orientation selectivity (OSI45, **Fig. 4B** and OSI90, data not shown) using previously published methods [7], [18], [19]. The proportion of visually-responsive neurons (showing a greater mean firing rate response for oriented gratings than for blank screen presentations) was similar between the main groups (Normal: 99.4%, VEH: 99.9%, ZAL: 99.2%, ESZ: 98.9%, TRA: 99.6%). We found that MD and subsequent sleep altered visual response properties in both NDE and DE pathways, consistent with prior reports of MD effects [20], [21]. For example, in VEH animals there were significant increases in peak firing rate and orientation selectivity of responses to stimuli presented to the NDE (relative to left eye responses in Normal cats; *p*<0.000001 and *p*<0.05, respectively, K-S test; **Fig. 4A–B**), and reductions in these parameters for DE responses (relative to Normal right eye responses; *p*<0.000001 and *p*<0.0005, respectively, K-S test).

In general, ESZ and ZAL tended to enhance changes in NDE and DE pathways, while TRA tended to inhibit these changes. For example, increased NDE peak firing and orientation selectivity (relative to Normal left eye responses) was observed in neurons from all hypnotic-treated animals (firing [**Fig. 4A**]: *p*<0.000001 for all groups vs. Normal, K-S test; OSI45 [**Fig. 4B**]: *p*<0.0005, *p*<0.000001, and *p*<0.05 for ZAL, ESZ, and TRA vs. Normal). However, relative to VEH, ZAL *further* increased NDE peak firing rates (*p*<0.0005 vs. VEH, K-S test), and ZAL and ESZ both further enhanced NDE OSI45 (*p*<0.05 and *p*<0.000001 vs. VEH, respectively). In contrast, TRA led to slightly (but not significantly, *p* = 0.09) reduced NDE peak firing rates relative to VEH, and reduced NDE OSI45 (*p*<0.05 vs. VEH). Similar effects of hypnotics on DE responses were observed. Neurons from all three treatment groups showed reduced DE peak firing rates and OSI45 (relative to right eye responses from Normal V1 neurons; firing [**Fig. 4A**]: *p*<0.000001, *p*<0.000001, and *p*<0.05 for ZAL, ESZ, and TRA; OSI45 [**Fig. 4B**]: *p*<0.000001, *p*<0.000001, and *p*<0.001 for ZAL, ESZ, and TRA). However, relative to VEH, ZAL *further* decreased DE peak firing rates (*p*<0.05), while TRA impaired DE response depression (*p*<0.000001 vs. VEH, K-S test). All three drugs also increased DE OSI45 relative to VEH (*p*<0.0005, *p*<0.000001, and *p*<0.005 for ZAL, ESZ, and TRA vs. VEH).

### Assessment of non-specific effects of trazodone on the visual cortex

Because TRA was the only compound to significantly affect ocular dominance plasticity, we conducted two additional sets of control experiments to rule out non-specific effects of TRA on visual cortical neurons (see **Text S1** for details). In the first set of experiments, we assessed the effects of TRA on ocular dominance in animals not exposed to prior MD (NoMD+TRA cats, *n* = 3). As shown in **Fig. S3A–D**, while TRA has clear effects on the sleep/wake architecture of these cats, it does not change scalar measures of ocular dominance. In the second set of experiments, we assessed the effects of acute administration of trazodone (vs. DMSO vehicle) in a subset of recorded neurons. This was done by recording a set of neurons (total *n* = 39) over a baseline period, for 30 min following i.v. administration of vehicle, and for 30 min following subsequent i.v. administration of trazodone, using procedures comparable to those employed previously by other investigators [2] and our own lab [3]. As shown in **Fig. S3E**, no significant effects of either treatment were found on peak firing rates, spontaneous firing rates, orientation selectivity, or visual responsiveness for this set of neurons.

## Discussion

Using a combination of polysomnography, *in vivo* single-unit recording, and intrinsic signal imaging, we assessed the effects of commonly-prescribed hypnotics on consolidation of ocular dominance plasticity during sleep. We found that the “z” hypnotics ZAL and ESZ have profound effects on sleep EEG activity, but only TRA - which antagonizes 5-HT_2_, H_1_, and α_1_-adrenergic receptors - significantly interferes with sleep-dependent consolidation of cortical plasticity.

### TRA effects on sleep-dependent consolidation of ocular dominance plasticity

We find that TRA impairs consolidation of cortical plasticity during post-MD sleep when administered at 10 mg/kg - a dosage roughly equivalent (on a mg/kg basis) to the highest clinical dose typically given to patients for insomnia and/or depression [22], [23]. In contrast, the dosages of ZAL and ESZ administered in this study (also 10 mg/kg) represent the upper limit used in many animal studies [24]–[26], and constitute between 30–100 times the maximum prescribed dose for humans [23], [27]. Thus the finding that 10 mg/kg TRA impairs sleep-mediated ocular dominance plasticity - while 10 mg/kg ZAL or ESZ do not significantly affect it - strongly indicates that pathways targeted by TRA at clinically-relevant doses are specifically involved in cortical remodeling during sleep.

It is unlikely that the effects of TRA on ocular dominance plasticity are caused by non-specific effects of TRA on V1 neurons. First, while the elimination half-life of TRA in healthy young subjects (∼5–6 h [28]) is longer than that of ZAL (about 1 h [29]), it is roughly equal to that of ESZ [27], [30], and is unlikely to be present at significant levels at the time of single-unit ocular dominance assessments, which typically took place 14–18 h after drug administration. Furthermore, prior studies examining the effects of 5-HT or α_1_-adrenergic receptor antagonism (antagonist properties associated with TRA) on response properties of V1 neurons reported no gross defects in visual processing [31]–[33]. We also find that while the compounds under study had different effects on *plasticity*, none of them grossly perturbed visual responses in V1 neurons (*e.g*., OSI45, peak firing rate; **Fig. 4**). Intrinsic signal angle and polar maps from TRA animals (**Fig. S2**) likewise show normal orientation tuning and response magnitude. And finally, we find no effects of TRA alone (without prior MD) on ocular dominance or visual responsiveness (**Fig. S3A–D**), and no acute effects of TRA administration on visual response properties of V1 neurons (**Fig. S3E**). Thus, the most parsimonious interpretation of our findings is that TRA directly interferes with synaptic plasticity mechanisms active in V1 during post-MD sleep.

While TRA did lead to substantial reductions in post-MD REM sleep (**Fig. 1**), this effect is unlikely to directly cause impairments in ocular dominance plasticity for the following reasons. First, similar reductions in REM were seen in all three hypnotic-treated groups, yet only TRA significantly impaired plasticity in V1. Indeed, a previous study showed that ocular dominance plasticity consolidation is not affected by administration of triazolam, although it caused a more profound suppression of REM than any of the drugs used in the current study [11]. Second, we have recently demonstrated that selective (non-pharmacological) REM sleep deprivation following MD does not impair consolidation of this plasticity [7]. Lastly, the degree of V1 plasticity during sleep is positively correlated with post-MD NREM - and not with REM - sleep [9].

It remains unclear, however, what aspects of NREM sleep are critical for consolidation of cortical plasticity. Cortical slow waves and spindle oscillations have been proposed as mediators of mnemonic processes [34], [35], as have cortical neurotransmission and neuromodulator release during NREM sleep [7], [36]–[39]. Our current findings suggest that TRA interferes with sleep dependent plasticity without affecting NREM cortical oscillations (**Fig. 2**). On the other hand, restorative sleep is reported following administration of “z” hypnotics which, as shown here, perturb NREM EEG activity [3], [4]. We find that these profound changes in NREM EEGs are not associated with inhibition of ocular dominance plasticity (**Fig. 2** and [11]). Together, the available data suggest that other cellular mechanisms active during NREM sleep - but not apparent at the level of the EEG - are critical for consolidating cortical plasticity.

Precisely what these mechanisms are is difficult to determine because TRA has many effects, including antagonism at 5-HT_2A_ and 5-HT_2C_, α_1_-adrenergic, and histamine H_1_ receptors. TRA also inhibits T-type calcium channels and is a weak serotonin reuptake inhibitor [14], [15], [40]. However, of these potential mechanisms, 5-HT_2_ receptor antagonism appears to be the most likely factor in our results. Selective intracortical blockade of 5-HT_2C_ receptors reduces ocular dominance plasticity [32], and 5-HT_2C_ receptors facilitate long-term synaptic potentiation and depression (LTP and LTD) in developing V1 *in vitro* [41], [42].

On the other hand, there is no evidence that histamine influences ocular dominance plasticity, and while noradrenergic signaling is critical for ocular dominance plasticity [43], its effects are mediated specifically by β-adrenergic receptors [44]. T-type calcium channels have been shown to influence ocular dominance plasticity [45], [46], but the affinity of TRA for these channels is much lower than for 5-HT_2_ receptors. At the dosage employed in our current studies (10 mg/kg, with a maximum effective concentration of around 25 µM), TRA is likely to profoundly antagonize 5-HT_2_ receptors but only modestly affect T-type channels [40], [47]. Lastly, selective serotonin reuptake inhibitors (*e.g*., fluoxetine) *augment* consolidation of motor skill learning during post-training sleep [38] and restore cortical plasticity in adult mice to levels seen during the critical period [48]. Therefore, our results are best explained by a blockade of 5-HT_2C_ receptors during sleep which impairs the normal strengthening and weakening of synapses in V1.

### Serotonin signaling during sleep and cortical plasticity

An activation of 5-HT_2_ receptors during sleep may seem counter-intuitive because, relative to waking, cortical serotonin levels are relatively low [49]–[51]). However, these concentrations may be sufficient to activate 5-HT_2C_ receptors, which have a relatively high affinity for serotonin compared to other 5-HT receptors [52], and which are maximally expressed in the visual cortex during the critical period [53]. It is also possible that serotonin release in the sleeping cortex can increase depending on prior waking experience. For example, locus coeruleus activity and cortical noradrenaline levels are specifically up-regulated during NREM sleep following training on an olfactory learning task, in a manner consistent with a role in memory consolidation [54], [55]. It is possible that a similar use-dependent activation of serotonergic pathways may also occur during sleep. Therefore, while the results of systemic injections should be cautiously interpreted, our findings suggest that blockade of 5-HT_2C_ receptors impairs the normal strengthening and weakening of synapses in V1 during post-MD sleep.

A serotonergic mechanism may also explain why, of the “z” hypnotics examined here and in a prior study, only zolpidem significantly reduces ocular dominance plasticity [11]. While this difference may be partly explained by minor differences in these drugs' affinity for α_1_-, α_5_- or γ_3_-containing GABA_A_ receptors [12], [13] or their pharmacokinetics [30] - *i.e*., by slightly differing GABA_A_ receptor agonist properties - it more likely reflects the known effects of zolpidem on cerebral serotonin signaling. At the dose used in our prior study, zolpidem has been shown to reduce cerebral serotonin accumulation [56] and enhance the suppression of firing in serotonin-releasing median raphe neurons by GABA_A_ receptor agonists [57]. In contrast, there is no evidence that the dosage of ZAL or ESZ used in our current experiment would have the same effect [58].

### Conclusions

The results of systemic drug treatment should be cautiously interpreted for several reasons. The effects of systemic drug treatments on central nervous system function may vary across mammalian species (*i.e.* cats vs. humans); therefore complementary studies in humans are required. In addition, our results do not exclude the possibility that supra-clinical doses of the “z” hypnotics might also inhibit the consolidation of cortical plasticity, or that alterations of monoaminergic signaling during wakefulness might impair the induction of ocular dominance plasticity.

Nevertheless, our findings are important because they provide new evidence suggesting that monoaminergic signaling during sleep contributes to cortical plasticity. In addition, and in agreement with an earlier report [11], they demonstrate that hypnotics producing major alterations in sleep architecture do not necessarily impair neural functions of sleep. Conversely, agents that produce more “physiological” sleep based on polysomnography - such as TRA - may grossly impair these functions.

Our results may also have important clinical implications. TRA is commonly prescribed to pediatric patients - not only for insomnia [59] and mood disorders [60], [61], but also for migraine prophylaxis [62] and treatment of behavior disorders (such as tic disorders [63], aggressive behavior disorders [64], and attention disorders [65]). Thus our current findings raise important questions regarding the consequences of long-term use of these drugs in pediatric populations.

## Materials and Methods

### Experimental design and formation of groups

Animals were housed and treated in accordance with University of Pennsylvania IACUC regulations for animal care and use. All invasive procedures were performed under anesthesia and survival procedures were accompanied with pre- and post-operative care as described previously [8], [9]. We used an experimental design similar to that used previously to test the effects of benzodiazepine and non-benzodiazepine hypnotics on sleep-dependent ocular dominance plasticity [11](**Fig. 1A**). At postnatal days P24–28, cats were randomly assigned to control (vehicle [VEH], *n* = 6; data from 4 cats reproduced with permission from Seibt et al. [11]) or experimental groups. Experimental groups were treated with either zaleplon (ZAL, *n* = 5), eszopiclone (ESZ, *n* = 6), or trazodone (TRA, *n* = 5), as described below (*Drug treatments*). Whenever possible, siblings were distributed evenly across different groups to minimize potential litter effects. In addition, a group of animals with unmanipulated, binocular vision and unmanipulated sleep (Normal, *n* = 7) was used for comparison of ocular dominance measures with the main treatment groups. Four of the Normal cats were previously used to provide normative data in an earlier study (reproduced with permission from Jha et al. [8]). There was no difference in age between any of the groups (*F* = 0.93, *p* = 0.46, one-way ANOVA). Circadian factors are unlikely to play a role in sleep-dependent ocular dominance plasticity because circadian rhythms in sleep/wake and other parameters are extremely weak in cats [66]–[68] and entirely absent in critical period kittens [69]. Nevertheless, all experimental manipulations were done at the same time of day.

### Surgical procedures and sleep/wake recording

All cats in VEH and hypnotic treatment groups were implanted with EEG/EMG electrodes on postnatal days P21–28 as described previously [11]. Briefly, five EEG electrodes were placed bilaterally in frontal and parietal bones of the skull (1–2 cm away from V1), and three EMG electrodes (braided stainless-steel wire) were placed deep into the nuchal muscle. Electrodes were connected to an electrical socket fixed to the skull with bone screws and dental acrylic. After 4–5 d of postoperative recovery, cats were placed in a light proof, illuminated sleep-recording chamber with a revolving base. EEG/EMG signals were continuously recorded during a 6-h baseline period, a 6-h monocular deprivation (MD) period, and a 6-h post-MD sleep period in total darkness (**Fig. 1A**). Cats were provided with food and water *ad lib* at all times. Polygraphic signals were amplified with an Astro-Med (West Warwick, RI) amplifier system, filtered (high-pass at 0.3 Hz, low-pass at 100 Hz), digitized at 200 Hz, and recorded as previously described using SleepSign software (Kissei Comtec; Irvine, CA)[8].

Following the baseline recording period, cats were anesthetized with isoflurane and had their right eyelids sutured closed as previously described [9]. Following recovery, cats were returned to their recording chambers and were kept awake (through a combination of gentle handling, novel object exploration, vocalization, and floor rotation) under normal room illumination for the next 6 h to provide a common stimulus for remodeling in V1 [8], [9]. The deprived eye remained sutured closed in all cases until the time of acute ocular dominance assessments.

### Sleep/wake analysis

EEG and EMG signals were used to assign polygraphic data into 8-s epochs of non-REM sleep (NREM), REM sleep, and waking states using SleepSign software. The proportion of time spent in REM, NREM, and waking (and mean bout duration for each state) was calculated separately for baseline, MD, and post-MD sleep periods using standard conventions. Fast-Fourier transforms (FFT; SleepSign) were used to assess EEG spectra between 0–40 Hz in each sleep state as described previously [11]. EEG power in each frequency band during post-MD sleep was normalized for each cat as a percentage of the pre-MD baseline. As described in the **RESULTS** section, we also observed an apparent intermediate state between waking and NREM sleep - described as “NREM-drowsy” (ND) - in a subset of ESZ- and ZAL-treated animals (*n* = 2 and *n* = 1, respectively). However, the amount of ND in these animals was very low across the 8-h post-MD sleep period (0.7%±0.4% and 2.1% of total recording time, respectively; data not shown). As described in a prior study [11], this sleep stage was analyzed separately for EEG power spectra but included in the sleep bout calculations as NREM because of its behavioral and EEG similarities to NREM sleep.

### Drug treatments

All drugs (and vehicle) were administered in a single i.p. injection which was given immediately following the 6-h period of waking MD, at the beginning of the post-MD sleep period (**Fig. 1A**). Zaleplon (ZAL; Sepracor; Marlborough, MA) and trazodone (TRA; Sigma; St. Louis, MO) were diluted in 0.25–0.75 ml of DMSO vehicle and administered at 10 mg/kg. Eszopiclone (ESZ; Sepracor) was diluted in 0.25–0.75 ml of an aqueous acetate buffer vehicle solution containing 28 mM glacial acetate and 221 mM sodium acetate trihydrate (pH 4.5), and delivered at a dosage of 1 mg/kg (*n* = 2 cats) or 10 mg/kg (*n* = 4 cats). Preliminary findings indicated that the low and high dosages of ESZ had similar effects on sleep and ocular dominance plasticity, and that neither of the two drug vehicles used as controls (DMSO, *n* = 5, or acetate buffer, *n* = 1) had substantial effects on sleep or ocular dominance plasticity. Thus, for simplicity, data from these animals were grouped together, in ESZ and VEH treatment groups, respectively.

### Single-unit electrophysiology and ocular dominance analysis

To assess ocular dominance and right eye/DE and left eye/NDE response properties, micro-electrode recordings of single neurons were performed in all groups as described previously [8]. Contact lenses were placed in the eyes (for optimum focus to a monitor positioned at a distance of 40 cm), and neuronal responses to grating stimuli presented to either eye were recorded using a 1×1 mm array of 16 electrodes (Frederick Haer; Bowdoinham, ME) which was placed within V1, as described previously [9]. Depth of recording (measured in 100 µM steps from the pial surface, as described previously [7]) was similar between neurons recorded from all hypnotic-treated groups and those recorded from VEH cats (*N.S.*, Dunn's *post hoc* test; median depth of recording sites [25^th^, 75^th^ percentile] for these groups were: VEH: 300 µM [200, 400 µM], ZAL: 200 µM [200, 400 µM], ESZ: 300 µM [200, 400 µM], TRA: 300 µM [200, 300 µM]). The number of neurons recorded from each animal also did not differ between groups (*N.S.*, one-way ANOVA; mean number of neurons recorded from these groups [±SEM] were: VEH: 115±12, ZAL: 131±13, ESZ: 129±15, TRA: 121±13). Thus there was no systematic bias in the distribution of recordings from the various groups across cortical layers. For each set of neurons recorded, eight full-field, slowly-drifting, reversing gratings (0.2 cycles/degree, 5-s presentation) were presented randomly to each eye four times (4×8 different orientations at 22.5° intervals+blank screen per eye).

Single-unit data was discriminated offline (Offline Sorter; Plexon, Inc.; Dallas, TX) and mean firing rates in each neuron were assessed for each eye and stimulus presentation as described previously [8]. Left eye/right eye response ratios were computed at the preferred orientation and ranked on a conventional 7-point ocular dominance scale using a computer algorithm that approximates subjective scoring rules [8]. Previously used and accepted scalar measures of ocular dominance were then used to quantify the single-unit distributions in each hemisphere [8], [9]. To simplify our presentation, the traditional contralateral bias index (CBI) was modified so that scores of 1 indicated complete dominance by the non-deprived eye, 0 complete deprived eye dominance and 0.5 equal representation of both eye in the hemisphere under study (a metric hereafter referred to as the “non-deprived eye bias index”: NBI). We also calculated monocularity indices (MI) for each hemisphere and for combined-hemisphere data, and shift indices (SI) as previously described [8], [9]. An MI value of 1 indicates a complete loss of binocular responses and 0 indicates that all neurons respond equally to stimuli presented to either eye. The shift index (SI) measures overall changes in ocular dominance across both hemispheres (CBI_Ipsilateral to the DE_- CBI_contralateral to the DE_) with a value of 0 indicating complete binocularity, and 1 or -1 indicating complete shifts toward one eye or the other [9], [70].

### Visual response properties

Several additional single-unit response properties were measured using previously published and established procedures [7]. For calculations of peak firing (at each neuron's preferred orientation) and to blank screen (a measure of spontaneous activity), firing rates were normalized to the mean firing rate of neurons recorded across the electrode array at a given recording position (across all stimulus presentations). This normalization, which is comparable to normalizations used elsewhere [71], corrected for normal fluctuations in anesthetic depth between measurements and inter-animal variability within a group. Orientation selectivity was calculated using the previously described orientation selectivity indices [18]. Briefly, mean firing rates were computed at each neuron's preferred stimulus orientation and the oblique (45° from preferred; OSI45) and orthogonal (90° from preferred; OSI90) orientation. Ratios of unit firing rates were calculated (response at oblique/preferred or orthogonal/preferred for OSI45 and OSI90, respectively) and subtracted from 1. Indices of 1 indicate a high degree of selectivity, and indices of 0 indicate a lack of selectivity. Because vehicle and hypnotic treatments similarly affected DE and NDE OSI45 and OSI90 values, OSI45 distributions only are shown for simplicity. Distributions of these response parameters were statistically evaluated for DE and NDE responses as described by others [19], [72]. For display purposes (*e.g.*, in **Fig. 4**) a running average smoothing function (sampling proportion  = 0.1; SigmaPlot; Systat Software, Inc.; San Jose, CA) was used in the cumulative histogram figures, but statistics were always performed on unsmoothed data.

### Statistics

All values are expressed as means±SEM as indicated. Statistical analyses were performed using SigmaStat software (Systat Software Inc.; San Jose, CA). All data were first tested for normality and skew. Parametric data were assessed using one- or two-way ANOVA and *post hoc* Holm-Sidak tests (SigmaStat software; Systat Software Inc.; San Jose, CA); in cases where non-parametric statistics were required, Kruskal Wallis ANOVA and Dunn's tests were used. Cumulative distributions of values for single-unit response properties (OSI, firing rates) were compared using Kolmogorov-Smirnov (K-S) tests as previously described [19], [72] (Matlab software; The Mathworks, Inc.; Natick, MA). Mean percentages and mean bout durations of sleep/wake data were either compared for the entire 8-h post-MD period (**Fig. 1**), or were analyzed using two-way repeated measures ANOVA to test for effects of drugs over time (**Fig. S1** **,** **Table S1**). EEG power spectra were compared separately in each 2-h bin in the post-MD period using two-way ANOVA (group and frequency as main factors).

## Supporting Information

Figure S1 Time course of hypnotic-induced sleep changes.
(5.02 MB TIF)

Figure S2 Drug effects on sleep-dependent ocular dominance plasticity: intrinsic signal imaging.
(4.45 MB TIF)

Figure S3 Assessment of non-specific effects of TRA on ocular dominance and visual responses.
(4.31 MB TIF)

Text S1 Supplemental Methods and Figure Legends
(0.06 MB DOC)

Table S1 (0.03 MB DOC)

Table S2 (0.03 MB DOC)
