# Supplementary figures and images for: The Sedating Antidepressant Trazodone Impairs Sleep-Dependent Cortical Plasticity
Source: PLoS One. 2009 Jul 1;4(7):e6078. doi: 10.1371/journal.pone.0006078 (PMC2699540; doi:10.1371/journal.pone.0006078)

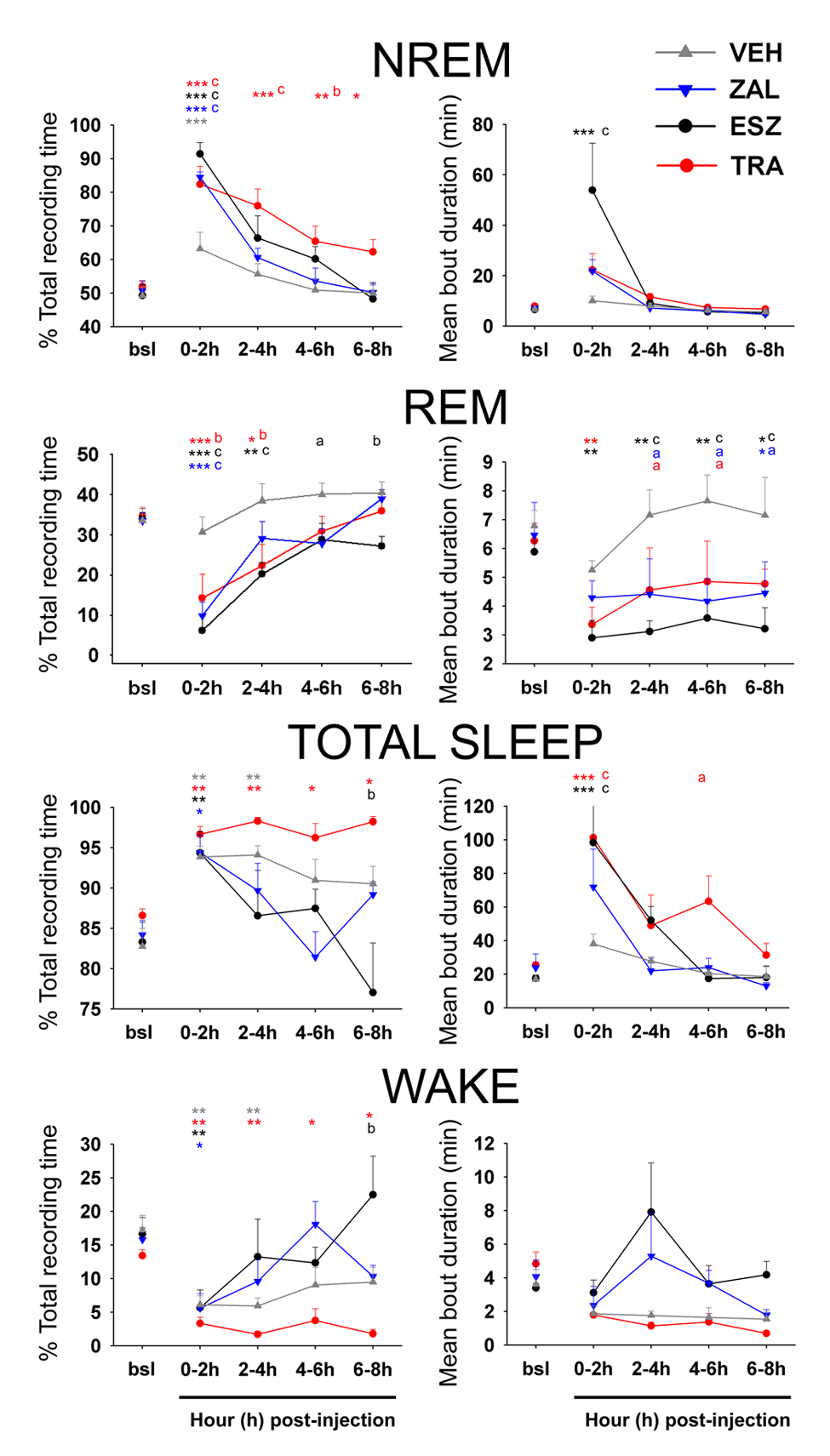

Supplement: Figure S1 — Time course of hypnotic-induced sleep changes. (5.02 MB TIF) [file pone.0006078.s001.tif]

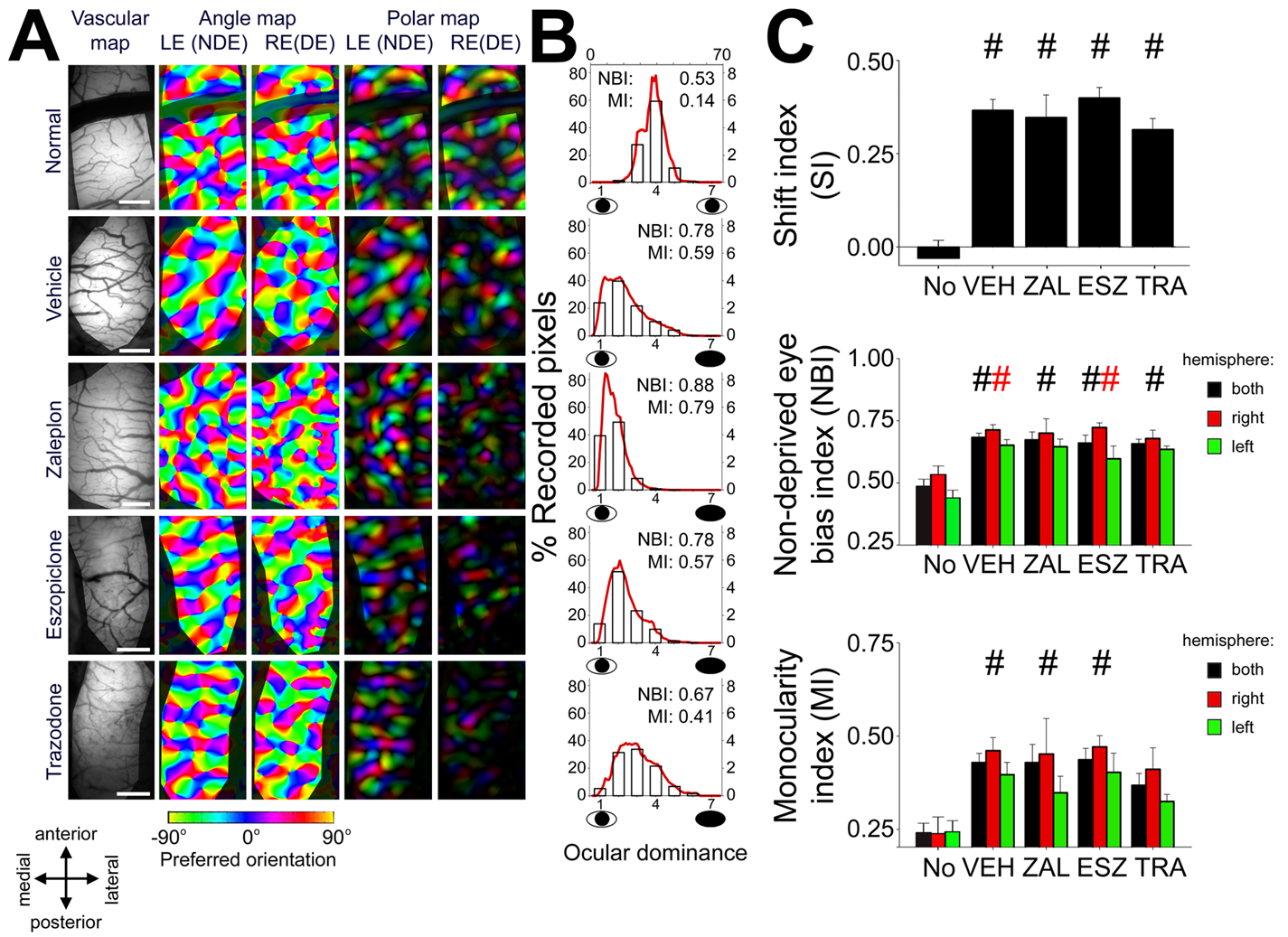

Supplement: Figure S2 — Drug effects on sleep-dependent ocular dominance plasticity: intrinsic signal imaging. (4.45 MB TIF) [file pone.0006078.s002.tif]

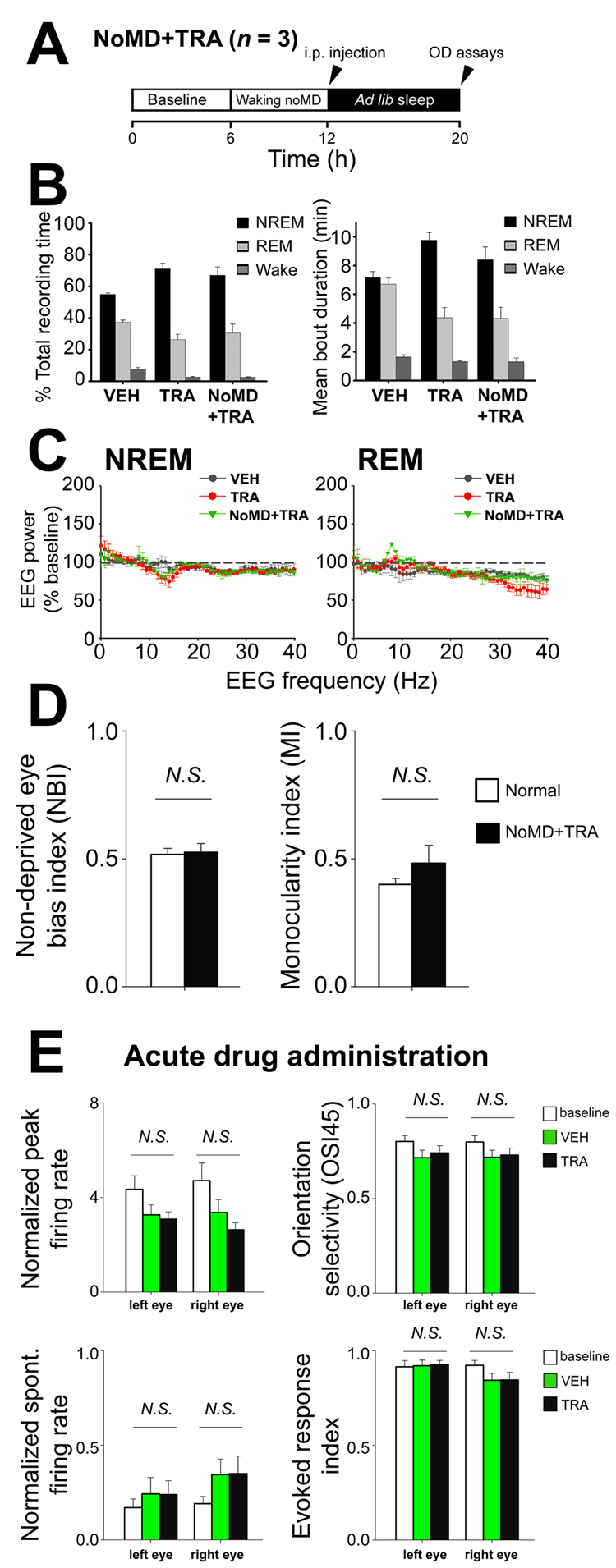

Supplement: Figure S3 — Assessment of non-specific effects of TRA on ocular dominance and visual responses. (4.31 MB TIF) [file pone.0006078.s003.tif]
