# Supplementary material for: The Sedating Antidepressant Trazodone Impairs Sleep-Dependent Cortical Plasticity
Source: PLoS One. 2009 Jul 1;4(7):e6078. doi: 10.1371/journal.pone.0006078 (PMC2699540; doi:10.1371/journal.pone.0006078)
Supplement: Text S1 — Supplemental Methods and Figure Legends (0.06 MB DOC) [file pone.0006078.s004.doc]

**Text S1.**

**Supplemental Methods**

*Intrinsic signal imaging and ocular dominance analysis*: Intrinsic signal imaging was performed using previously described methods [1,2]. The skull and dura over right and left V1 were removed, and intrinsic signals were recorded using a CCD camera (Dalstar 1M30; Dalsa; Waterloo, ON) focused 600 µM below the pial surface. Responses were measured for slowly rotating full-field grating stimuli, presented to either the re-opened right, deprived eye (DE) or left, non-deprived eye (NDE). Images of the cortical surface were continuously acquired at a rate of 30 frames/s and saved after temporal (4 frames) and spatial (2 × 2 pixels) binning. We continuously monitored the physiological state of each cat during imaging to ensure that heart-rate and expired CO_2_ levels were always comparable between left eye and right eye presentations. We also examined a number of representative pixel responses in every map (as described in Kalatsky et al., 2003) to verify that the mapping signal was not obscured by vasomotor, respiratory, or other potential noise sources.

Intrinsic signal maps were constructed as described previously [2]. Following correction for hemodynamic delay as described in Kalatsky et al. (2003), maps generated for each eye were cropped to remove vascular artifacts. Stimulus-induced luminance changes at each pixel were then used to create orientation preference (angle) maps. Ocular dominance at each pixel was determined by computing a distribution of left eye/right eye pixel response ratios which were subsequently collapsed into a more traditional 7-point ocular dominance scoring system (with 4 corresponding to equal response for both eyes, and 1 and 7 corresponding to the contralateral and ipsilateral eye, respectively [2]. As described for single-unit measures, previously used and accepted scalar measures of ocular dominance (SI, MI, NBI; see **MATERIALS AND METHODS**) were then used to quantify the pixel distributions in each hemisphere [2,3].

*Supplemental control experiments: isolation of non-specific effects of TRA:* Two additional sets of control experiments were performed to test for non-specific TRA effects on sleep-dependent plasticity. First, we determined whether TRA alone changed ocular dominance when administered prior to normal sleep. Three cats (NoMD+TRA, **Fig. S3A**; age at recording = 33.7 ± 1.2 d) were treated exactly as TRA cats, but did not undergo MD. Instead they were kept awake for 6 h with normal, binocular vision, administered TRA (10 mg/kg in DMSO) i.p., and then allowed to sleep *ad lib* in complete darkness for the next 8 h (to match procedures used in TRA cats). Single-unit measurements of ocular dominance were subsequently recorded from V1 of these cats and compared with those of Normal cats (**Fig. S3D**). Scalar ocular dominance measures were compared between Normal and NoMD+TRA cats using Student's *t*-tests.

To assess acute effects of TRA on ocular dominance, firing rate, and visual response properties in V1 (**Fig. S3 E-F**), TRA (10 mg/kg in DMSO) or (for comparison) DMSO vehicle were administered i.v. to the three NoMD+TRA kittens following ocular dominance assessment, using procedures comparable to those employed previously by other investigators [4] and our own lab [5]. The experiment began with a baseline recording using the single-unit stimulus set described in **MATERIALS AND METHODS**. Recordings were then made for the same subset of neurons 30 min following VEH administration, and 30 min after subsequent TRA administration. Only neurons which could be reliably discriminated for all three phases of the experiment (based on electrode position, waveform, amplitude, and orientation preference characteristics) were included in analysis. Visual response parameters were assessed for neurons during each phase of the experiment as described in **MATERIALS AND METHODS**. To test for effects of VEH and TRA on visual responsiveness (**Fig. S3**), an additional response parameter was assessed. An evoked response index (ERI) [6] was calculated for both eyes by subtracting the ratio of mean firing rate during blank screen presentation / mean firing rate at the preferred stimulus (VR [2]) from 1 (*i.e*., 1-[spontaneous background rate/maximal evoked rate]). Neurons with ERI scores ≤ 0 are considered not visually responsive (*i.e.*, evoked firing equal to or less than firing during blank screen presentations); ERIs for visually responsive neurons are thus positive values with a maximum of 1. This measure is similar to previously-used measurements of signal-to-noise in critical period cat V1 [7]. The acute effects of VEH and TRA on visual response properties were statistically assessed with Friedman’s repeated measures ANOVA followed by Holm-Sidak *post hoc* tests where appropriate.

**Figure S1.** ***Time course of hypnotic-induced sleep changes*.** NREM**,** REM, total sleep (NREM+REM)**,** and wake amounts and mean bout durations for each group are shown at baseline (bsl) and in 2-h bins during the post-MD sleep period. The left column shows mean (± SEM) state amounts as a % of total recording time and the right column shows corresponding mean (± SEM) bout duration in minutes during the post-MD period. Mean (± SEM) baseline (bsl) values did not differ between groups for any of the baseline values (one-way ANOVA, *N.S.*). Differences in sleep/wake parameters between groups as a function of time during post-MD sleep were assessed with a repeated-measures two-way ANOVA (results shown in **Table S1**). Levels of statistical significance are reported for each group individually on the graphic. ***** indicates *p* < 0.05, ****** indicates *p* < 0.001, and ******* indicates *p* < 0.0001, vs. bsl, respectively. **^a^** indicates *p* < 0.05, **^b^** indicates *p* < 0.001, and **^c^** indicates *p* < 0.0001, vs. VEH, respectively, Holm-Sidak *post hoc* test.

**Figure S2. *Drug effects on sleep-dependent ocular dominance plasticity: intrinsic signal imaging*.** Representative maps of intrinsic signal responses from each of the treatment groups are shown in **A**. Maps are from hemispheres ipsilateral to the right (deprived) eye (DE). The leftmost column shows maps of surface vasculature above primary visual cortex (V1). The highlighted area in all maps corresponds to the cortical region that was optimally focused and free of vascular artifacts. Scale bar = 1 mm. The second and third columns show orientation preference (angle) maps generated by stimuli presented to the left eye (or non-deprived eye; LE/NDE) and the right eye (or deprived eye RE/DE), respectively. The fourth and fifth columns are corresponding polar maps. For both angle and polar maps, color corresponds to the preferred stimulus orientation at each pixel (key shown below). For polar maps, pixel brightness represents the magnitude of the response driven by each eye. Pixel-by-pixel ocular dominance histograms (**B**) were computed for each map by comparing the relative NDE and DE response magnitudes at each imaged pixel. The pixel distribution was collapsed into a classic 7-point ocular dominance measurement scale [8] as described [2]. In all histograms, an ocular dominance score of 1 indicates pixel responses exclusively driven by the left eye (NDE), 7 indicates pixel responses driven exclusively by the right eye (DE), and 4 represents pixels responding equally to both eyes. Values to the right are the corresponding non-deprived eye bias index (NBI; top) and monocularity index (MI; bottom) scores for each map. Quantitative measurements of ocular dominance for the main groups are shown in **C**. Significant effects of treatment group were found for SIs (*F* = 16.3, *p* < 0.001), NBIs (NBI_both hemispheres_: *F* = 7.9, *p* < 0.001; NBI_right hemisphere_: *H* = 11.1, *p* = 0.026; NBI_left hemisphere_: *N.S.*, one-way ANOVA), and MIs (MI_both hemispheres_: *H* = 18.6, *p* < 0.001; MI_right hemisphere_: *N.S.*; MI_left hemisphere_:*N.S.*, one-way ANOVA). Though SI and NBI values tended to be lower for TRA-treated animals than for VEH, ZAL, and VEH groups, similar effects vs. Normal (No) were seen for all of these groups (**#** indicates *p* < 0.05 vs. No, Dunn's or Holm-Sidak *post hoc* tests). MI values, however, were not significantly different between Normal and TRA cats.

**Fig. S3. TRA administration to cats with normal vision does not affect ocular dominance and does not acutely affect visual response properties.** To determine if TRA alone grossly perturbed the visual cortex we administered TRA (10 mg/kg in DMSO) to cats with normal vision. (**A**) Experimental design. NoMD+TRA cats were treated exactly as cats given TRA after MD, but binocular vision was left intact prior to the *ad lib* sleep period. (**B** and **C**) Like cats given TRA after MD, NoMD+TRA cats showed increased NREM sleep and decreased REM sleep and wake relative to VEH cats (VEH and TRA data are replicated from **Fig. 1** for comparison; **B**), and showed no changes in EEG power density relative to VEH (data in **C** show EEG spectral power calculated over the entire 8-h post-sleep period). (**D**) Scalar measures of ocular dominance (combined-hemisphere NBIs and MIs) were similar between single neurons recorded from NoMD+TRA cats (*n* = 665) and those recorded from Normal cats (data replicated from **Fig. 3**; Student's *t*-test, *N.S.*). The proportion of visually-responsive neurons recorded was similar between NoMD+TRA and Normal conditions (99.7% and 99.4% of recordings, respectively). (**E**) Acute effects of TRA were assessed by measuring ocular dominance and visual response properties in a subset of recorded neurons during baseline, 30 min after VEH administration, and 30 min after subsequent TRA administration. (**E**) Neither VEH nor TRA had a significant effect on peak firing rate at the preferred stimulus orientation, spontaneous firing rate during blank screen presentation, orientation selectivity, or visual responsiveness (mean values ± SEM shown separately for left and right eyes; *N.S.*, Friedman’s repeated measures ANOVA). NBIs and MIs for recorded neurons were similar in NoMD+TRA cats across baseline, VEH administration, and TRA administration (mean ± SEM of 0.50 ± 0.04 and 0.40± 0.04 during baseline, 0.50 ± 0.02 and 0.36 ± 0.03 at 30 min post-VEH administration, and 0.58 ± 0.05 and 0.39 ± 0.04 at 30 min post-TRA administration, *N.S.*, one-way repeated measures ANOVA).

1. Kalatsky VA, Stryker MP (2003) New paradigm for optical imaging: temporally encoded maps of intrinsic signal. Neuron 38: 529-545.

2. Jha SK, Jones BE, Coleman T, Steinmetz N, Law C, et al. (2005) Sleep-dependent plasticity requires cortical activity. Journal of Neuroscience 25: 9266-9274.

3. Frank MG, Issa NP, Stryker MP (2001) Sleep enhances plasticity in the developing visual cortex. Neuron 30: 275-287.

4. Higley MJ, Contreras D (2007) Cellular mechanisms of suppressive interactions between somatosensory responses In Vivo. J Neurophysiol 97: 647-658.

5. Aton SJ, Seibt J, Dumoulin M, Jha SK, Steinmetz N, et al. (2009) Mechanisms of sleep-dependent consolidation of cortical plasticity. Neuron 61: 454-466.

6. Seibt J, Aton S, Jha SK, Dumoulin M, Coleman C, et al. (2008) The non-benzodiazepine hypnotic Zolpidem impairs sleep-dependent cortical plasticity. SLEEP 31: 1381-1391.

7. Lodovichi C, Berardi N, Pizzorusso T, Maffei L (2000) Effects of Neurotrophins on Cortical Plasticity: Same or Different? JNeurosci 20: 2155-2165.

8. Hubel DH, Wiesel TN (1970) The period of susceptibility to the physiological effects of unilateral eye closure in kittens. J Physiol 206: 419-436.
