# Supplementary material for: The Sedating Antidepressant Trazodone Impairs Sleep-Dependent Cortical Plasticity
Source: PLoS One. 2009 Jul 1;4(7):e6078. doi: 10.1371/journal.pone.0006078 (PMC2699540; doi:10.1371/journal.pone.0006078)
Supplement: Table S1 — (0.03 MB DOC) [file pone.0006078.s005.doc]

**Table S1. *Two-way ANOVA results for effects of drug treatment and time on % of total recording time (% TRT) and mean bout duration for each vigilance state.***

| ***state*** | ***% TRT/ bout duration*** | ***effect of group*** | ***effect of time*** | ***group x time interaction*** |
| --- | --- | --- | --- | --- |
| **NREM** | %TRT | *F* = 6.2,  *p* < 0.01 | *F* = 50.4,  *p* < 0.001 | *F* = 2.7,  *p* < 0.01 |
|  | bout duration | *F* = 2.5,  *p* = 0.09 | *F* = 12.3,  *p* < 0.001 | *F* = 2.9,  *p* < 0.005 |
| **REM** | %TRT | *F* = 7.3,  *p* < 0.005 | *F* = 26.1,  *p* < 0.001 | *F* = 2.3,  *p* < 0.05 |
|  | bout duration | *F* = 6.8,  *p* < 0.005 | *F* = 5.4,  *p* < 0.001 | *F* = 0.8,  *p* = 0.63 |
| **total sleep** | %TRT | *F* = 6.5,  *p* < 0.005 | *F* = 8.6,  *p* < 0.001 | *F* = 2.2,  *p* < 0.05 |
|  | bout duration | *F* = 4.1,  *p* < 0.05 | *F* = 18.0,  *p* < 0.001 | *F* = 1.8,  *p* = 0.076 |
| **wake** | %TRT | *F* = 6.6,  *p* < 0.005 | *F* = 8.9,  *p* < 0.001 | *F* = 2.1,  *p* < 0.05 |
|  | bout duration | *F* = 2.7,  *p* = 0.07 | *F* = 2.3,  *p* = 0.07 | *F* = 1.1,  *p* = 0.33 |
