# Supplementary material for: The Sedating Antidepressant Trazodone Impairs Sleep-Dependent Cortical Plasticity
Source: PLoS One. 2009 Jul 1;4(7):e6078. doi: 10.1371/journal.pone.0006078 (PMC2699540; doi:10.1371/journal.pone.0006078)
Supplement: Table S2 — (0.03 MB DOC) [file pone.0006078.s006.doc]

**Table S2. *Two-way ANOVA results for effects of drug treatment and time on cortical EEG spectral power.***

| ***state*** | ***time (h post-MD)*** | ***effect of group*** | ***group x frequency interaction*** |
| --- | --- | --- | --- |
| **NREM** | 0-2 h | *F* = 542.0, *p* < 0.001 | *F* = 4.2, *p* < 0.001 |
|  | 2-4 h | *F* = 314.2, *p* < 0.001 | *F* = 3.4, *p* < 0.001 |
|  | 4-6 h | *F* = 335.8, *p* < 0.001 | *F* = 2.9, *p* < 0.001 |
|  | 6-8 h | *F* = 227.2, *p* < 0.001 | *F* = 2.6, *p* < 0.001 |
| **REM** | 0-2 h | *F* = 137.2, *p* < 0.001 | *F* = 1.8, *p* < 0.001 |
|  | 2-4 h | *F* = 217.8, *p* < 0.001 | *F* = 1.6, *p* < 0.001 |
|  | 4-6 h | *F* = 209.0, *p* < 0.001 | *F* = 1.4, *p* = 0.001 |
|  | 6-8 h | *F* = 216.9, *p* < 0.001 | *F* = 1.8, *p* = 0.001 |
